# Green satsuma mandarin orange (*Citrus unshiu*) extract reduces adiposity and induces uncoupling protein expression in skeletal muscle of obese mice

**DOI:** 10.1007/s10068-018-0503-1

**Published:** 2018-11-02

**Authors:** Jeong Kee Kim, Hyun Woo Jeong, A Young Kim, Yong Deog Hong, Ji Hae Lee, Jin Kyu Choi, Jae Sung Hwang

**Affiliations:** 1 Department of Genetic Engineering and Graduate School of Biotechnology, College of Life Sciences, Kyung Hee University, 1732 Deogyeong-daero, Giheung-gu, Yongin-si, Gyeonggi-do 17104 Republic of Korea; 2 Vital Beautie Research Division, Amorepacific R&D Center, 1920 Yonggu-daero, Giheung-gu, Yongin-si, Gyeonggi-do 17074 Republic of Korea; 3 QA Team, Aestura Corporation, Gongdan1-ro 36, Ansung-si, Gyeonggi-do 17575 Republic of Korea

**Keywords:** Green mandarin orange, Hesperidin, UCP3, Adiposity, Muscle

## Abstract

Increased fat mass, which is induced by the storage of excess nutrients, is considered a causal factor for various metabolic disorders, including insulin resistance, type 2 diabetes, hyperlipidemia, hyperglycemia, hypertension, atherosclerosis, and non-alcoholic fatty liver disease. Therefore, it is necessary to prevent hyperadiposity to sustain a healthy life. Recently, uncoupling proteins (UCPs) were suggested to be molecular targets for curing obesity and its complications. In this study, green satsuma mandarin orange (*Citrus unshiu*) extract (GME) increased UCP3 expression in cultured myocytes. In a diet-induced obese animal model, administration of GME reduced fat mass and average fat cell size. Similar to in vitro experiments, GME restored expression of UCP3 in skeletal muscle. Moreover, GME also induced UCP2 expression in skeletal muscle. In conclusion, GME is suggested to be a novel functional dietary supplement for adiposity control through induction of UCPs.

**Electronic supplementary material:**

The online version of this article (10.1007/s10068-018-0503-1) contains supplementary material, which is available to authorized users.

## Introduction

Input and output of energy are tightly regulated to maintain energy homeostasis. When energy homeostasis is imbalanced as a result of either increased calorie intake or decreased energy consumption, excess nutrients are stored in the adipose tissue thereby causing obesity (Kopelman, 2000). Since increased fat mass is a risk factor of various metabolic disorders, including hyperglycemia, type 2 diabetes, hepatic steatosis, hyperlipidemia, hypercholesterolemia, hypertension, atherosclerosis, and cardiovascular diseases (Kopelman, 2000), controlling energy balance and adiposity is important to prevent or treat metabolic syndromes and extend health span.

ATP is mainly synthesized in mitochondria at the expense of stored nutrients such as carbohydrates and lipids. Through the electron transport chain, a proton gradient is created in the intermembrane space of the mitochondria. ATP synthase produces ATP by transporting protons from the intermembrane space to the matrix. Uncoupling proteins (UCPs), proton channels located in the mitochondrial inner membrane, dissipate the proton gradient to generate heat instead of ATP (Ricquier et al., 1999). Through the generation of heat, UCPs enable survival of the organism by maintaining the body temperature. From the viewpoint of energy metabolism, activation of UCPs can boost energy consumption to reduce fat content (Ricquier et al., 1999) by competing with ATP synthase for use of accumulated protons (Ricquier, 2017). As UCP activation boosts energy consumption to reduce fat content, it is feasible that UCPs might be targets of anti-obesity therapy.

Flavonoids, a class of plant-derived secondary metabolites, exert a number of beneficial effects on energy metabolism. For example, flavonoids have been reported to exhibit anti-allergic, anti-inflammatory, antioxidant, anti-microbial, and anti-cancer activities (Cazarolli et al., 2008; Cushnie and Lamb, 2005; Cushnie and Lamb, 2011; Friedman, 2007). Furthermore, flavonoid-rich extracts from various plants are also reported to relieve obesity and related complications (Kamisoyama et al., 2008; Kwon et al., 2017; Vernarelli and Lambert, 2017; Wu et al., 2010; Xu et al., 2014; Yang et al., 2017). Accordingly, flavonoid supplementation might be an anti-obesity therapy to prevent or treat metabolic disorders, including obesity. Among various plant-derived flavonoid-rich extracts, we found that green mandarin orange extract (GME), an ethanol-insoluble precipitation of unripe fruit of *Citrus unshiu*, contains a large amount of hesperidin and is capable of inducing UCP expression. Therefore, we hypothesized that GME might be useful to control adiposity.

In this study, we evaluated the efficacy of GME for adiposity reduction. GME augmented UCP3 expression in cultured myocytes. Concordant with the in vitro result, GME increased UCP3 expression in skeletal muscle in a diet-induced obese animal model. Additionally, expression of UCP2 in skeletal muscle was also increased in vivo by GME administration. As a result, fat mass and average adipocyte size of GME-treated mice were decreased in spite of a high-fat diet. Taken together, our data suggest that GME might be used as anti-adiposity therapy.

## Materials and methods

### Reagents and GME preparation

Hesperidin was purchased from Tokyo Chemical Industry (Tokyo, Japan). Trifluoroacetic acid (TCA) and methanol were purchased from Sigma (St. Louis, MO, USA). For the preparation of GME, unripe fruit of *Citrus unshiu* was obtained from the orchards of Jeju Island (Korea) and extracted with ethanol two times. Briefly, the dried unripe fruit was extracted with 4 L of 70% aqueous ethanol three times at 80 °C for 1 h, followed by further extraction with 95% aqueous ethanol to yield 90% ethanol-insoluble precipitate (GME). For analysis of the hesperidin content in GME, an HPLC (LC-2000Plus, Jasco International, Tokyo, Japan) assay using a Luna^®^ C18 5 μm column (4.6 × 250 mm) was performed. A gradient elution was achieved with various ratios of solvent A (0.1% TCA in water) to solvent B (methanol), with a flow rate of 1 mL/min as follows: 0–25 min, 70:30, 26–30 min, 10:90, and 31 min onward, 70:30. The wavelength of acquisition was set to 280 nm. The average content of hesperidin in the GME was 35% (Supplemental Fig. 1).

### Cell culture

C2C12 mouse myocytes were purchased from American Type Culture Collection (Manassas, VA, USA) and grown in Dulbecco’s Modified Eagle Medium (DMEM, Sigma) supplemented with 10% fetal bovine serum (HyClone, Logan, UT, USA). Myocytes were differentiated as described earlier (Jeong et al., 2011b). Briefly, confluent C2C12 cells were maintained with DMEM supplemented with 2% horse serum (HyClone) for 7 days. During myocyte differentiation, the medium was changed daily. All media were supplemented with 100 units/mL of penicillin and 100 mg/mL of streptomycin (Sigma), and cells were maintained at 37 °C in a humidified atmosphere containing 5% CO_2_.

### Analysis of gene expression

RNA was isolated using TRIzol^®^ reagent (Thermo Fisher Scientific, Waltham, MA, USA), following the manufacturers protocol. Each RNA sample (2 μg) was subjected to reverse transcription for cDNA synthesis (RevertAid First Strand cDNA Synthesis Kit, Thermo Fisher Scientific). Relative mRNA levels were determined by quantitative real-time PCR (qPCR, Bio-RAD CFX96, Bio-rad, Hercules, CA, USA) using the QuantiSpeed SYBR One-step kit (PhileKorea, Seoul, Korea) and appropriate primers (Bioneer, Daejeon, Korea). The sequences of primers used in qPCR assay are provided in Supplemental Table 1.

### Animal experiment

The animal experiment was adhered to OECD guidelines and approved by the Aestura Institutional Animal Care and Use Committee (IACUC17-012). Male, 7-week-old C57BL/6 mice were purchased from Samtako Bio Korea (Osan, Korea). During animal experiment, animals were maintained on a controlled humidity of 50–60%, temperature of 21–25 °C, and 12-h light/dark cycle. After adaptation, mice were randomly divided into three groups (normal diet [ND], high-fat diet [HFD], and HFD-GME; n = 9 per group). The HFD and HFD-GME groups were fed a HFD (60% calories from fat, Research Diet, New Brunswick, NJ, USA), whereas the ND group was fed the AIN-76A diet (Research Diet) for 10 weeks. During the experiment, GME (75 mg/kg; dissolved in distilled water) was orally administered once a day. The ND and HFD groups were given vehicle (distilled water) instead of GME. Body weight and food intake were measured weekly.

After the animal experiment, mice were fasted overnight and sacrificed. Gonadal white adipose tissue (WAT) and skeletal muscle tissues were prepared. The WAT was weighed and then immediately fixed with formalin. Following formalin fixation, tissues were paraffin-coated, sectioned, and stained with hematoxylin–eosin for analysis of tissue histology. Skeletal muscle tissue was used to analyze mRNA expression of UCPs.

### Statistical analysis

All values are the means of at least triplicate samples. Error bars represent standard deviation (for in vitro experiments) and standard error (for in vivo experiment), respectively. A *P* value < 0.05, as calculated by one-way ANOVA followed by Tukey honest significant difference, was regarded as statistically significant.

## Results and discussion

### GME augments mRNA expression of uncoupling protein 3 in cultured myocytes

**Fig. 1:**
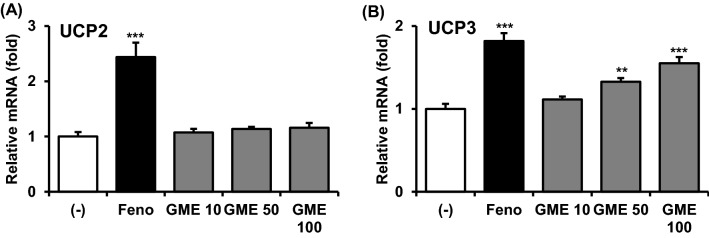
GME increases mRNA expression of genes related to lipid metabolism in cultured cell lines. Differentiated C2C12 myocytes were incubated with or without GME (10, 50, and 100 μg/mL, respectively) for 24 h. mRNA expression of UCP2 (**A**) and UCP3 (**B**) was measured by qPCR analysis and normalized to cyclophilin expression. ***P < 0.001 versus (-), **P < 0.01 versus (-)

By reducing the proton pool that is used to generate ATP, activation of UCP leads a robust increase in energy consumption. To determine whether GME increases UCP expression, we treated differentiated C2C12 myocytes with GME. Unexpectedly, GME failed to induce UCP2 expression [Fig. 1(A)] but increased mRNA expression of UCP3 [Fig. 1(B)], a muscle-specific isoform, in a dose-dependent manner. Thus, GME specifically augmented UCP3 expression in cultured muscle cells.

### GME reduces body fat content

**Fig. 2:**
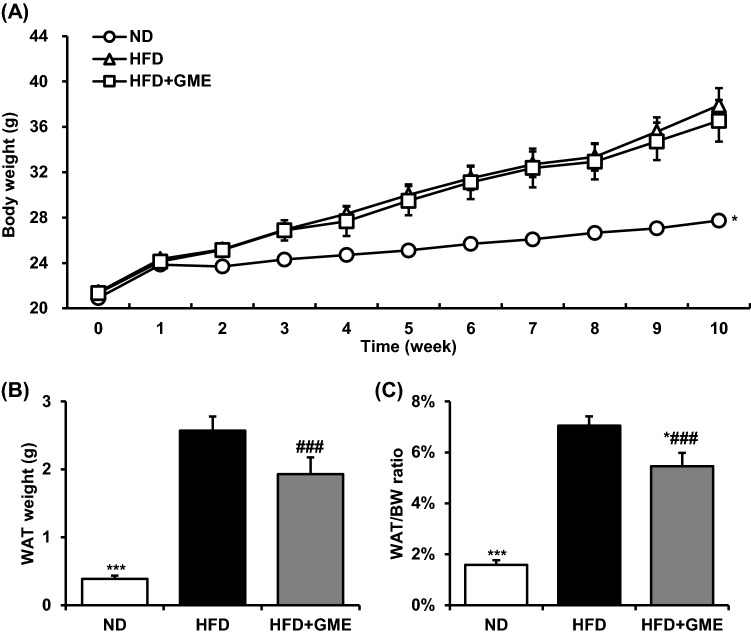
GME reduces fat mass regardless of body weight change. (**A**) Body weight change during animal experiment, ( **B**) weight of WAT, and (**C**) WAT mass/body weight ratio. ^###^*P* < 0.001 versus ND, ***P < 0.001 versus HFD, *P < 0.05 versus HFD

We observed that GME induced expression of UCP3 in differentiated myocytes. To determine whether GME exerts a beneficial effect on obesity, we conducted an in vivo experiment using mice fed a high-fat diet. During the animal experiment, the average daily food intake was not significantly changed (data not shown). Contrary to expectations, a significant change in body weight was not observed [Fig. 2(A)]; however, the weight of WAT was slightly decreased by GME administration [Fig. 2(B), *P* = 0.076]. As a result, the fat mass/body weight ratio, which represents the adiposity, was significantly reduced in the HFD-GME group, compared with that of the HFD group [Fig. 2(C)]. These data indicate that GME would be a useful agent for adiposity control.

### GME decreases average fat cell size and restores UCP expression

**Fig. 3:**
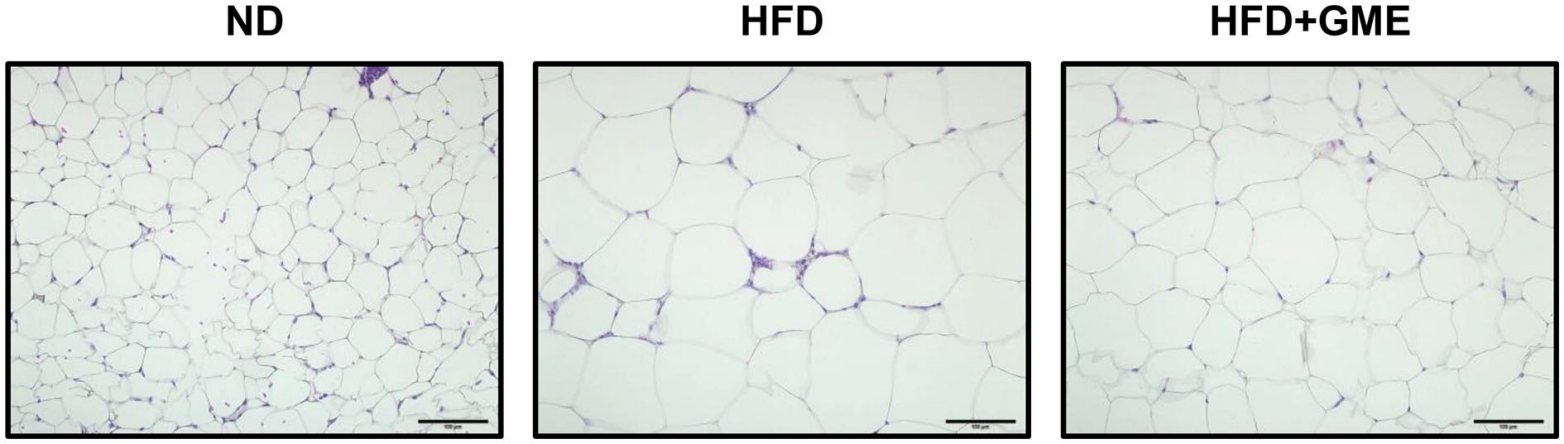
GME decreases average adipocyte size. Tissue histology data of WAT. Reduced average size of adipocytes was observed in the GME-treated high-fat diet group (right panel) compared with obese mice (middle panel)

Enlarged adipocytes contain a large amount of lipids, which can trigger inflammatory responses and immune cell infiltration thereby provoking insulin resistance (Cooke et al., 2016; Nguyen et al., 2007; Xu et al., 2003). Although GME failed to reduce body weight, GME administration alleviated the excessive gain of adiposity in diet-induced obese mice (Fig. 2). To confirm whether GME reduces adipocyte size in WAT, we measured the average size of adipocytes in WAT. Concomitant with the reduction of adipose tissue mass, GME also decreased the average size of adipocytes (Fig. 3).

**Fig. 4:**
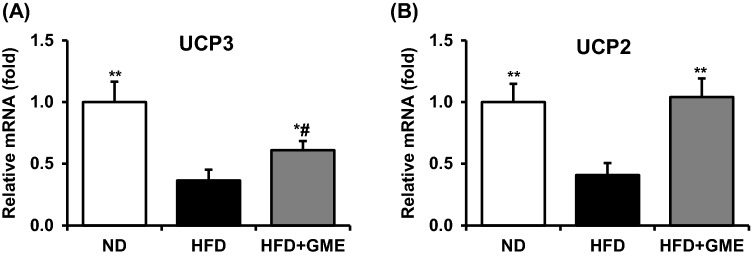
GME restored the HFD-mediated reduction of UCP2 and UCP3 expression in skeletal muscle. Relative mRNA expression of UCP3 (**A**) and UCP2 (**B**) in skeletal muscle was measured by qPCR analysis and normalized to cyclophilin expression. ^#^*P* < 0.05 versus ND, **P < 0.01 versus HFD, *P < 0.05 versus HFD

Next, we measured mRNA expression of UCP3 in skeletal muscle to check whether GME induces UCP3 expression. Similar to the data for cultured myocytes, GME rescued the suppression of UCP3 expression in skeletal muscle [Fig. 4(A)]. Interestingly, mRNA expression of UCP2 was also induced by GME administration in skeletal muscle [Fig. 4(B)], in contrast to cultured myocytes [Fig. 1(A)]. Based on these data, it is likely that GME exerts beneficial effects on energy metabolism by inducing UCP expression in skeletal muscle.

### Hesperidin mediates the effect of GME on the induction of UCP3 expression

**Fig. 5:**
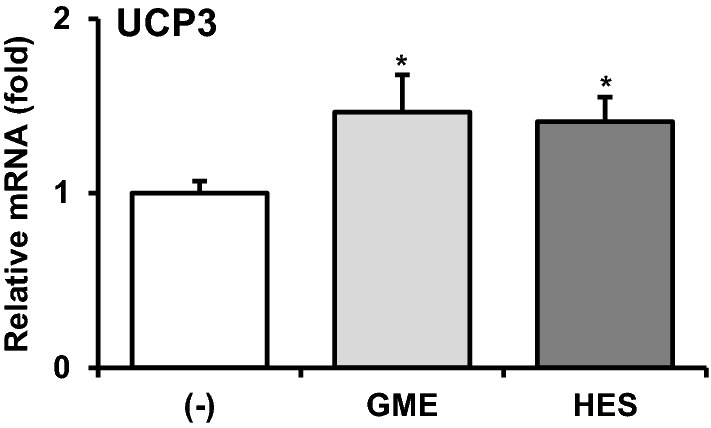
Hesperidin mediates the effect of GME on UCP expression. Differentiated C2C12 myocytes were treated with GME (100 μg/mL) or hesperidin (HES; 35 μg/mL) for 24 h. mRNA expression of UCP3 was evaluated by qPCR experiment and normalized to cyclophilin expression. *P < 0.05 versus (-)

As mentioned above, GME contains a large amount of hesperidin (35%). To determine whether hesperidin mediates the effect of GME on UCP3 induction, we treated myocytes with GME (100 μg/mL) and hesperidin (35 μg/mL; an equivalent amount to the hesperidin content in GME) and compared the mRNA expression level of UCP3. Interestingly, the effect of hesperidin on UCP3 expression was similar to that of GME (Fig. 5).

To make sure that hesperidin is the only effector of GME on UCP3 expression, we prepared fractionated flavonoids from GME (Supplemental Fig. 1). As expected, only one fraction which contains hesperidin increased mRNA expression of UCP3 in C2C12 cells (Supplemental Fig. 2). Based on these data, it is feasible that hesperidin mediates effect of GME on the induction of UCP3 expression in skeletal muscle.

The “energy wasting” feature of UCPs leads cells to consume more energy source to meet the ATP demands. Therefore, enhancing UCP expression could be a feasible approach to reduce fat content by promoting energy catabolism. For this reason, UCPs have recently been extensively investigated for the treatment of obesity and its related complications. For instance, Supaglutide, a novel glucagon-like peptide-1 analogue, is reported to exhibit an anti-obesity effect through the regulation of UCP1 (Wan et al., 2017). In addition, sea algae are suggested to induce thermogenesis and prevent obesity-induced cardiovascular dysfunction (Grasa-Lopez et al., 2016). Moreover, the combination of resveratrol and quercetin is reported to upregulate UCP2 expression in the fat tissue of rats with metabolic syndrome (Castrejon-Tellez et al., 2016). Conversely, animals lacking UCP2 expression are reported to be vulnerable to inflammatory responses (Bai et al., 2005; Sun et al., 2011). Therefore, the agonism of UCPs might provide benefits for obesity and related metabolic disorders that are closely associated with oxidative stress and inflammatory responses (Xu et al., 2003). As an inducer of UCP2 and UCP3, GME could be considered as a potential treatment for chronic inflammation and/or oxidative stress–mediated metabolic disorders, and not only for adiposity control.

Unlike other anti-obesity supplements, GME reduced fat mass without changing body weight. This result indicates that augmentation of the mass of other tissue(s) would counterbalance the weight loss effect of GME. Interestingly, the weight of liver and other weighable tissues was not increased (data not shown). Although we did not measure the weight of skeletal muscle, it is likely that the restoration of UCP expression and enhancement of lipid metabolism might be the causal factor of enlarged skeletal muscle. Indeed, hesperetin, an aglycone form hesperidin, is proposed to promote myocyte differentiation (Jeong et al., 2011a), indicating that GME would contribute to enhanced generation and energy metabolism of skeletal muscle. Further studies are required to reveal the effect and action mechanism of GME on muscle metabolism.

Hesperidin is one of the representative citrus flavonoids with numerous beneficial effects on energy metabolism (Assini et al., 2013), and the most abundant ingredient of GME. Hesperidin supplementation improved obesity-related metabolic parameters in obese animal models (Ferreira et al., 2016; Lu et al., 2013). Furthermore, hesperidin in combination with caffeine was reported to ameliorate weight gain in animal and human studies (Ohara et al., 2015; Ohara et al., 2016). Previous studies indicated that hesperidin treatment—alone or combination with other components—exhibits an anti-obesity effect. In our study hesperidin mediated the induction of UCP expression by GME, indicating that hesperidin could be an effector molecule of GME for adiposity control. Regarding the definition of obesity, our results imply that GME containing hesperidin possesses anti-obesity properties, and might be especially useful for the treatment of abdominal obesity.

Interestingly, in skeletal muscle GME augmented mRNA expression of UCP2 in addition to UCP3, which was not observed in cultured myocytes. The GME-mediated upregulation of UCP2 expression in skeletal muscle would be an indirect effect of GME, possible through the metabolism of hesperidin and inter-organ crosstalk. For example, enzyme or microorganism-mediated modification of hesperidin would yield hesperetin or other types of hesperidin metabolites, and some of the various hesperidin derivatives might be causal factors for UCP2 induction. Because these modifications mainly occur in the gut or liver during digestion, additional effects of GME due to hesperidin metabolites will not be observed in cultured cell lines. Nonetheless, we assume that UCP3 would be a primary effector of GME as UCP3 is abundantly expressed in skeletal muscle (Supplemental Fig. 3). Although the differential UCP expression pattern between in vitro and in vivo experiments cannot be explained at this time, our fundamental finding is that GME possesses a regulatory effect on UCPs to relieve excessive gains of adiposity. Future studies will reveal the mechanism by which UCP2 expression is regulated by GME administration.

In summary, GME ameliorated excess gain of adiposity in diet-induced obese mice without a change in food intake. As the adiposity decreased, the average size of adipocytes also decreased. In skeletal muscle, GME upregulated mRNA expression of UCP which is reported to promote lipid consumption. Based on our data, we propose GME as a novel regulator of adiposity for the treatment of obesity and related metabolic complications.

## Electronic supplementary material

Below is the link to the electronic supplementary material.

Supplementary material 1 (DOCX 18 kb)

Supplemental Fig. 1. Analysis of GME. The composition of flavonoids in GME was determined by HPLC analysis described in Materials & Methods. The content of each flavonoids in GME is 0.3% (nobiletin), 2.8% (tangeretin), 35% (hesperidin), and 2.2% (5,6,7,4’-tetramethoxyflavone), respectively, and the fractions containing each flavonoid were prepared for further analysis described in Supplemental Fig. 2. Supplemental Fig. 2. Effect of flavonoids in GME on UCP3 expression. Diffrentiated C2C12 myocytes were treated with GME (100 μg/ml) or flavonoid-containing fractions (equivalent concentration to the composition of each flavonoid in 100 μg/ml of GME) which were prepared in Supplemental Fig. 1 for 24 h. mRNA expression of UCP3 was evaluated by qPCR experiment and normalized to cyclophilin expression. N-F; nobiletin-containing fraction, T-F; tangeretin-containing fraction, H-F; hesperidin-containing fraction, M-F; 5,6,7,4’-tetramethoxyflavone-containing fraction. ** P < 0.01 vs. (-), * P < 0.05 vs. (-). Supplemental Fig. 3. Comparison of mRNA expression level of UCP2 and UCP3 in skeletal muscle. Relative expression level of UCP2 and UCP3 was analyzed by qPCR and normalized to cyclophilin expression. *** P < 0.001 vs. UCP2. (PPTX 233 kb)
