# Supplementary material for: Green satsuma mandarin orange (Citrus unshiu) extract reduces adiposity and induces uncoupling protein expression in skeletal muscle of obese mice
Source: Food Sci Biotechnol. 2018 Nov 2;28(3):873–9. doi: 10.1007/s10068-018-0503-1 (PMC6484071; doi:10.1007/s10068-018-0503-1)
Supplement: Supplementary file 1 — Supplementary material 1 (DOCX 18 kb) [file 10068_2018_503_MOESM1_ESM.docx]

**Supplemental Table 1.** Sequences of primers used for qPCR analysis.

| Genes | 5’ Sequence | 3’ Sequence |
| --- | --- | --- |
| Cyclophilin | CAGACGCCACTGTCGCTTT | TGTCTTTGGAACTTTGTCTGCAA |
| UCP2 | GCCACTTCACTTCTGCCTTC | GAAGGCATGAACCCCTTGTA |
| UCP3 | TGGCCCAACATCACAAGAAAT | ACGCAGAAAGGAGGGCACAAAT |
