# Supplementary material for: Green satsuma mandarin orange (Citrus unshiu) extract reduces adiposity and induces uncoupling protein expression in skeletal muscle of obese mice
Source: Food Sci Biotechnol. 2018 Nov 2;28(3):873–9. doi: 10.1007/s10068-018-0503-1 (PMC6484071; doi:10.1007/s10068-018-0503-1)
Supplement: Supplementary file 2 — Supplemental Fig. 1. Analysis of GME. The composition of flavonoids in GME was determined by HPLC analysis described in Materials & Methods. The content of each flavonoids in GME is 0.3% (nobiletin), 2.8% (tangeretin), 35% (hesperidin), and 2.2% (5,6,7,4’-tetramethoxyflavone), respectively, and the fractions containing each flavonoid were prepared for further analysis described in Supplemental Fig. 2. Supplemental Fig. 2. Effect of flavonoids in GME on UCP3 expression. Diffrentiated C2C12 myocytes were treated with GME (100 μg/ml) or flavonoid-containing fractions (equivalent concentration to the composition of each flavonoid in 100 μg/ml of GME) which were prepared in Supplemental Fig. 1 for 24 h. mRNA expression of UCP3 was evaluated by qPCR experiment and normalized to cyclophilin expression. N-F; nobiletin-containing fraction, T-F; tangeretin-containing fraction, H-F; hesperidin-containing fraction, M-F; 5,6,7,4’-tetramethoxyflavone-containing fraction. ** P < 0.01 vs. (-), * P < 0.05 vs. (-). Supplemental Fig. 3. Comparison of mRNA expression level of UCP2 and UCP3 in skeletal muscle. Relative expression level of UCP2 and UCP3 was analyzed by qPCR and normalized to cyclophilin expression. *** P < 0.001 vs. UCP2. (PPTX 233 kb) [file 10068_2018_503_MOESM2_ESM.pptx]

## Slide 1
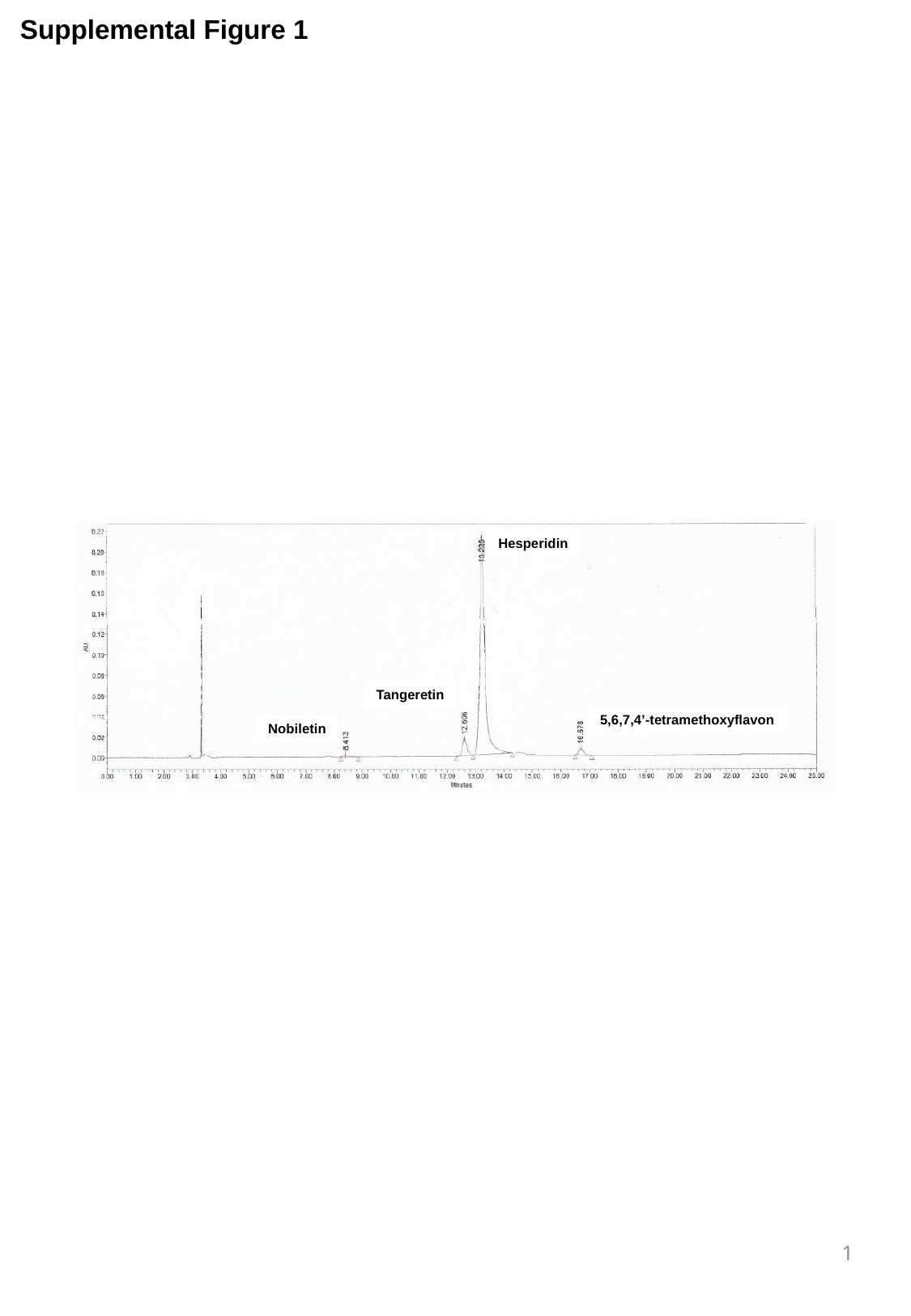

Supplemental Figure 1
Hesperidin
Tangeretin
5,6,7,4’-tetramethoxyflavon
Nobiletin
20

## Slide 2
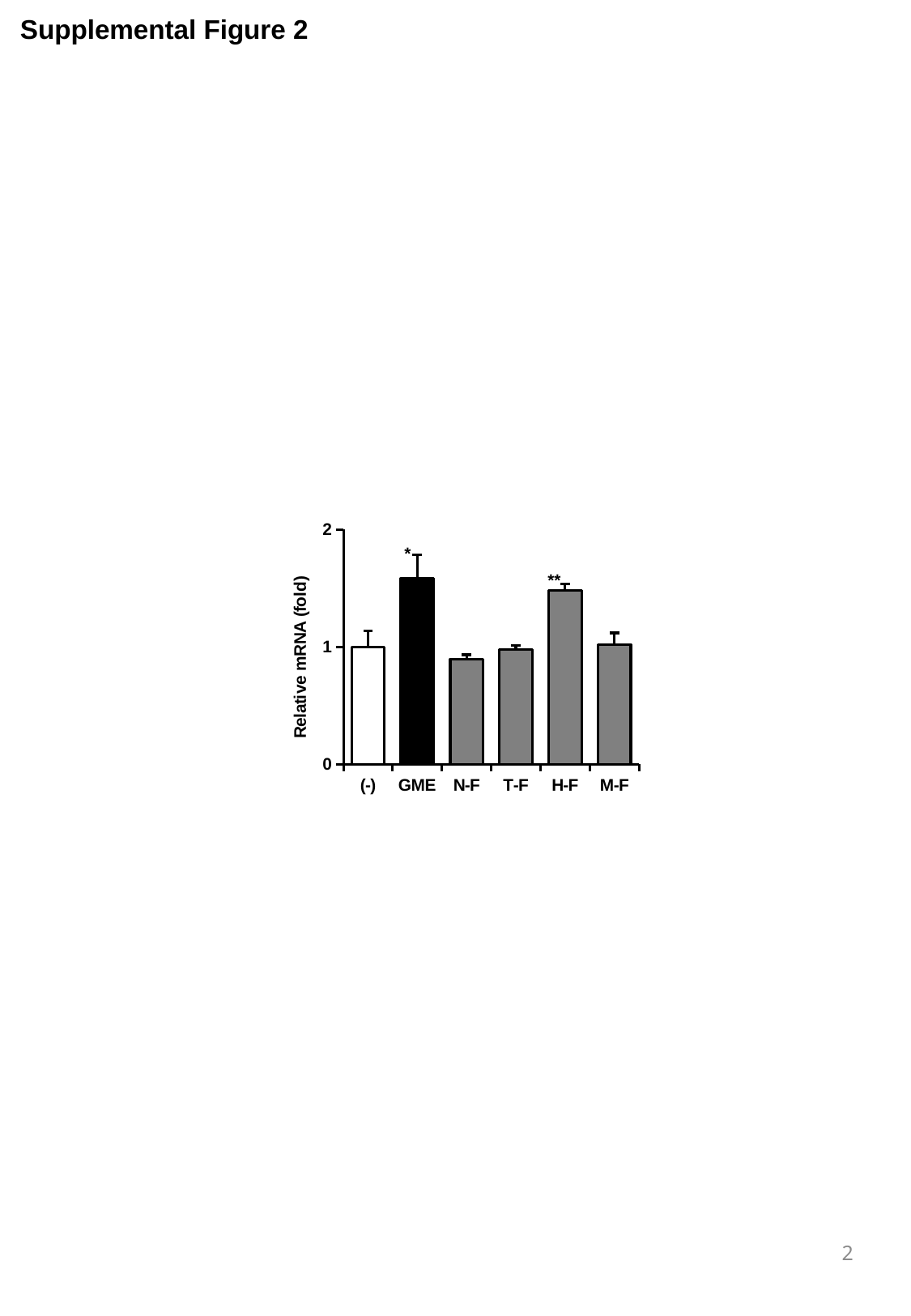

Supplemental Figure 2
### Chart
| Category | |
|---|---|
| (-) | 1.0 |
| GME | 1.5816596305052664 |
| N-F | 0.8939478315743026 |
| T-F | 0.978098575233423 |
| H-F | 1.479215110274302 |
| M-F | 1.0198858247043852 |*
**
21

## Slide 3
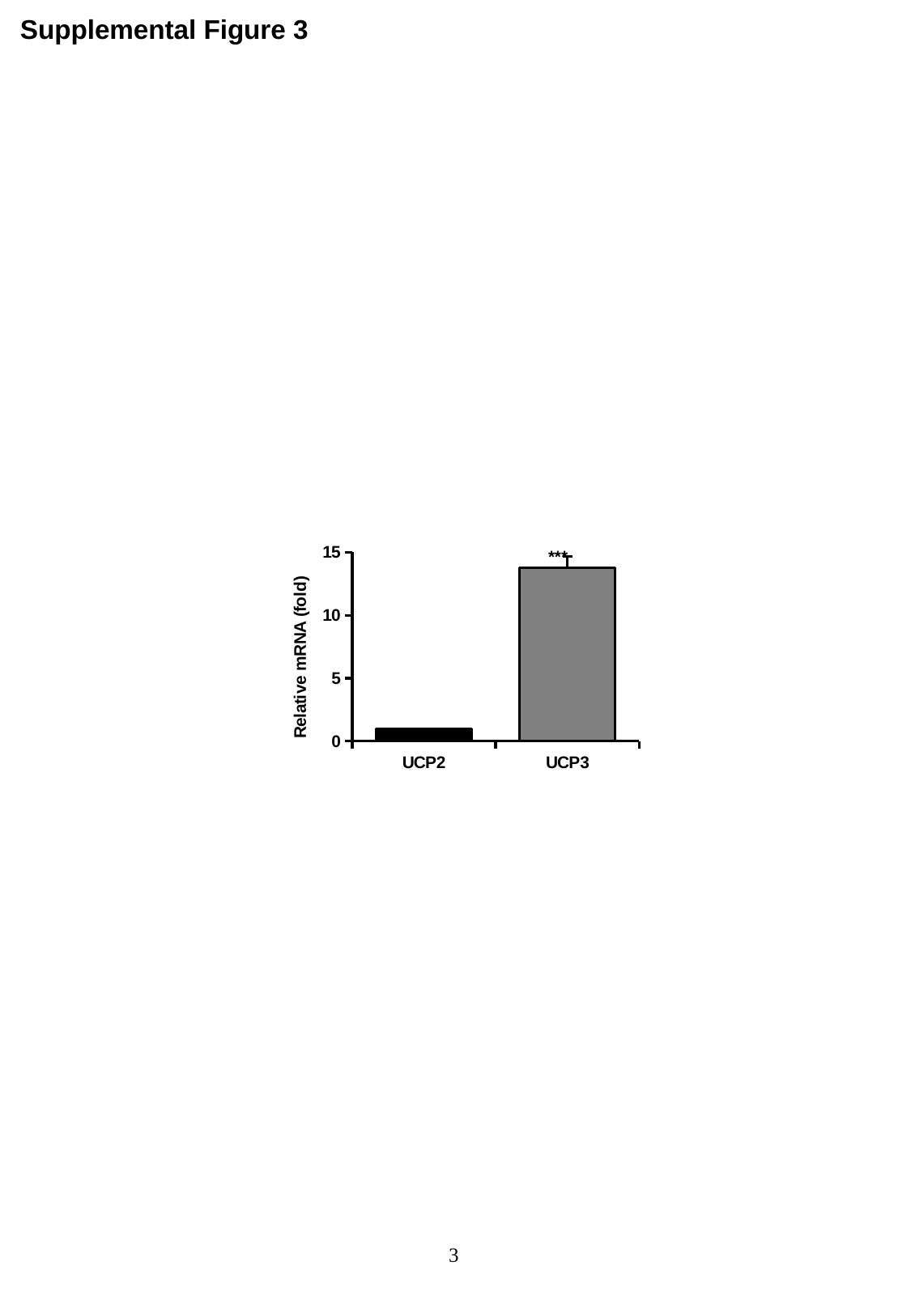

Supplemental Figure 3
### Chart
| Category | |
|---|---|
| UCP2 | 1.0 |
| UCP3 | 13.756422128289834 |***
22
